# Bucillamine‐induced interstitial pneumonitis

**DOI:** 10.1002/jgf2.169

**Published:** 2018-03-30

**Authors:** Keitaro Nakamoto, Yoshiaki Tanaka, Yuka Sasaki, Hajime Goto

**Affiliations:** ^1^ Department of Respiratory Medicine Japan Anti‐Tuberculosis Association Fukujuji Hospital Tokyo Japan

**Keywords:** : bucillamine, interstitial pneumonitis

## Abstract

We diagnosed as bucillamine‐induced interstitial pneumonitis and were able to observe changes in pulmonary lesions and therapeutic effects by using HRCT.

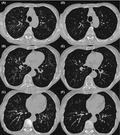

A 77‐year‐old woman was admitted to hospital due to cough lasting for a month. She was diagnosed as having rheumatoid arthritis (RA) 10 years ago and had been taking bucillamine for two months. Vital signs were normal with body temperature 36.8°C, respiratory rate 20/min, heart rate 68/min, blood pressure 86/57 mm Hg, and oxygen saturation 97% measured on ambient air.

Chest X‐ray showed bilateral nodular opacities (Figure 1). High‐resolution computed tomography (HRCT) showed ground‐glass opacities bilaterally along the bronchovascular bundles, bronchiectasis, and bronchial wall thickening (Figure 2A–C). Laboratory examination showed elevation of surfactant protein D (156 ng/mL). Pulmonary function testing showed a decline in her % predicted value for diffusing capacity of the lung carbon monoxide to 61.7%. Bronchoalveolar lavage fluid showed a slight increase in lymphocytes and decline of the CD4/8 ratio to 0.2. Just before she visited our hospital, no abnormal shadows were pointed out on chest X‐ray. Furthermore, we found that she had used bucillamine 10 years ago, and the same symptoms and abnormal chest shadows had occurred after using it then. At that time, her symptoms and the images had improved only with the discontinuation of bucillamine. On the basis of this coincidental discovery following the re‐administration of bucillamine, we diagnosed her as having bucillamine‐induced interstitial pneumonitis. We discontinued administration of the bucillamine and began treatment with oral prednisolone. Her symptoms and findings on HRCT promptly improved (Figure 2D–F).

**Figure 1:**
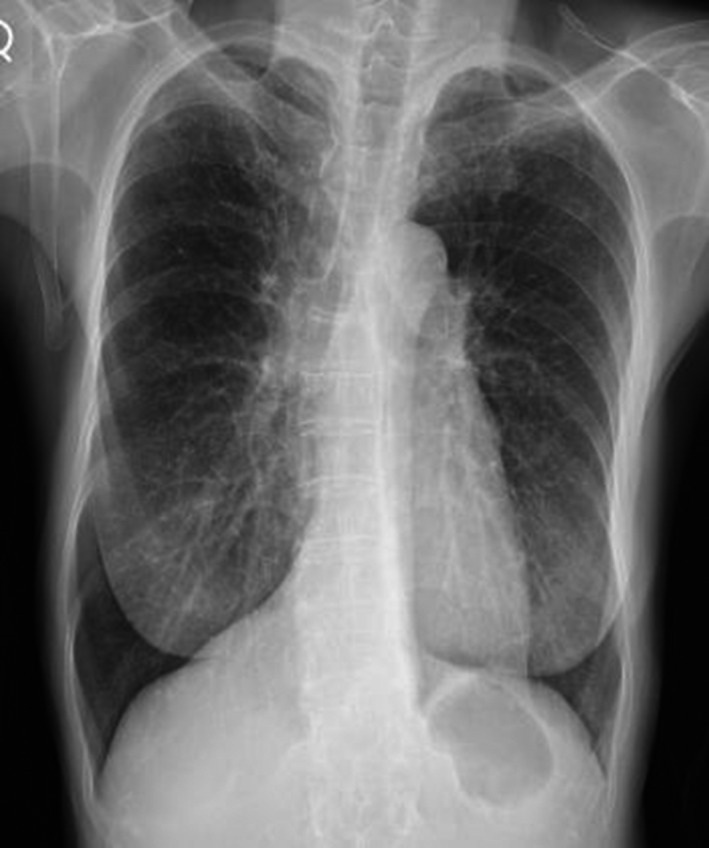
Chest X‐ray on the day of admission showed bilateral nodular opacities

**Figure 2:**
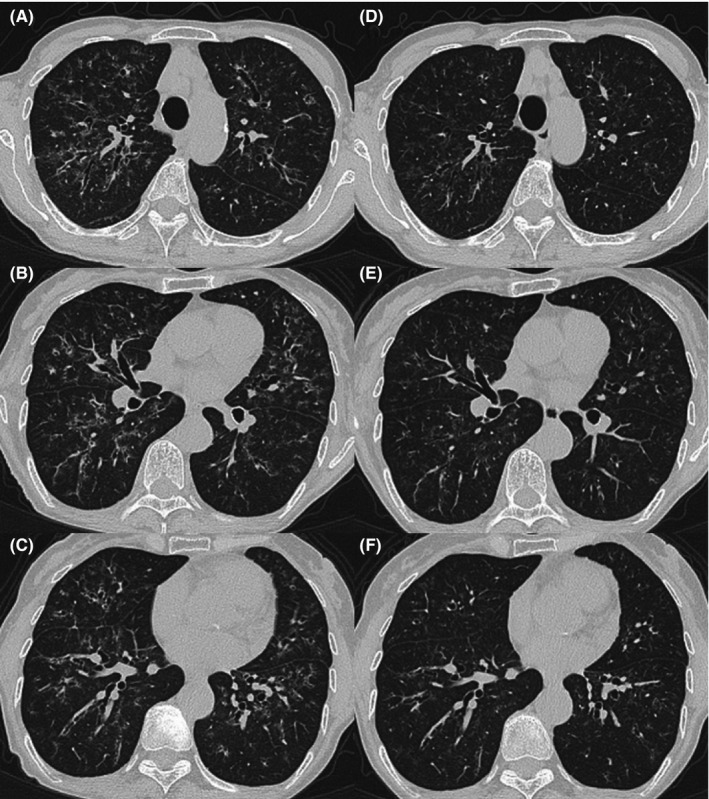
Chest CT on the day of admission showed ground‐glass opacities bilaterally along the bronchovascular bundles, bronchiectasis, and bronchial wall thickening (A–C). After treatment, these findings improved (D–F)

Bucillamine, a D‐penicillamine analog, is one of the disease‐modifying drugs for rheumatoid arthritis and is available in Japan and South Korea.^1^, ^2^ There are few reports of interstitial pneumonitis due to bucillamine, especially with HRCT findings. Inokuma et al reported that nine of 13 patients showed patchy mottled infiltrates in the bilateral center but not in the periphery on chest X‐ray.^3^ Another report showed bilateral diffuse ground‐glass opacities along the bronchovascular bundles and thickening of the interlobular septa.^4^ In the present case, we were able to observe changes in pulmonary lesions and therapeutic effects more precisely than in previous reports using HRCT with 1‐mm slice thickness.

## CONFLICT OF INTEREST

The authors have stated explicity that there are no conflicts of interest in connection with this article.
